# Medical News and Miscellany

**Published:** 1888-03

**Authors:** 


### Medical News and Miscellany.

Small Pox.—We regret to learn that our excellent friend Dr. G. W. Christian. of Burnet, has small pox in his family, in the person of his sister-in-law, Mrs. Love.

Congratulations: Dr. R. M. Swearingen was made unusually happy on the 8th instant by the advent in his family of an interesting little *grand daughter*, born to Mr. and Mrs. E. B. Robinson. As it is not a boy, we insist that it bear the name of the honored grandfather—a name too illustrious and good to be permitted to expire by limitation.

---

MARRIED in Greenville, Texas, February 15, 1888, Dr. W. S. Nobles of Collinsville, to Miss Ida Chandler, daughter of Dr. M. M. Chandler, of Greenville. At the wedding both Dr. Nobles and Dr. Chandler subscribed for "Daniel's Texas Medical Journal" through the Journal's staunch friend, Dr. F. E. Yoakum, President of the East Line Medical Association.

---

MARRIED at LaGrange, Texas, Wednesday, March 7, 1888, Dr. R. H. Eanes, of Taylor, to Miss Irene Hill, of LaGrange. No cards. We extend our hearty congratulations to our friend, the doctor, and with him all the happiness he anticipates.

---

MARRIED.—Dr. W. D. Palten, of Van Alstyne, to Miss Belle Short, of San Angelo, January 12, 1888.

---

THE PAPERS read at the AUSTIN DISTRICT MEDICAL SOCIETY on the 8th, together with a full report of the interesting discussions following each one, will be published in the next (April) number, and, as usual, we shall get out an extra-edition of that number, the last one preceeding the big medical convention.

---

EPISTAXIS.—Dr. H. C. Ghent calls attention to the value of dilute cider vinegar as a remedy in obstinate nose bleed.

---

DR, J. B. STINSON, of Sherman, and Dr. M. Farrer, of Ft. Worth are at the New York Polyclinic.

---

DR. M. HAGGARD, late of Buffalo, has removed to Mountain Peak; Dr. T. P. Davis, of Dublin, has removed to Canton, and Dr. J. F. McCarty, of Canton has removed to Dublin.

---

DR. TALLIAFERRO, of Roger's Prairie, has removed to Bryan; Dr. A. A. Lyon, of Tyler, has removed to Shreveport, La.

---

SOUTH-WESTERN UNIVERSITY M. E. CHURCH SOUTH, OR "THE UNIVERSITY OF SAN ANTONIO." with a Medical Department. We learn just as we go to press, that the foundation of a University, comprising a Faculty of Sciences, of Laws, of Theology and of Medicine in San Antonio has been determined upon by the M. E. Church South, with ample means for the erection of necessary buildings and equipments. The late hour at which we get the information precludes the expression of opinion on, or the giving of details of the Medical feature. We hope to discuss this very important move in the next issue.

